# Light-Sheet Fluorescence Imaging Reveals Three-Dimensional Amyloid Burden Reduction Following Five Weeks of Swimming Exercise in Alzheimer’s Mouse

**DOI:** 10.3390/ijms26031249

**Published:** 2025-01-31

**Authors:** Hye Joo Son, Suk Hyun Lee

**Affiliations:** 1 Department of Nuclear Medicine, Dankook University Medical Center, Dankook University College of Medicine, Cheonan, Chungnam 31116, Republic of Korea; 2 Department of Radiology, Hallym University Kangnam Sacred Heart Hospital, Hallym University College of Medicine, Seoul 07441, Republic of Korea

**Keywords:** Alzheimer’s disease, amyloid, exercise, light-sheet illuminating fluorescence microscope, tissue clearing

## Abstract

Emerging evidence from observational studies suggests that lifestyle modifications, particularly moderate-intensity exercise, may confer neuroprotective benefits against dementia, potentially by enhancing brain resistance through clearance mechanisms. Using light-sheet fluorescence microscopy (LSFM) with tissue clearing, we investigated the role of voluntary swimming in ameliorating β-amyloid pathology in a transgenic Alzheimer’s disease (AD) mouse model. Twenty 52-week-old hAPPsw mice were randomly divided into a 5-week voluntary swimming intervention group and a control group (each *n* = 10). Each session included a 10-min swim followed by a 10-min rest, escalating from one session per day in the first week to three sessions per day by the fifth week. The excised brains were prepared using tissue-clearing and volume immunostaining with thioflavin-S for β-amyloid. For LSFM imaging, the individual plaque area and volume, total plaque load, and morphological parameters were quantified via an Imaris-based three-dimensional (3D) volumetric surface model. Visual comparison revealed that the intervention group presented significantly lower β-amyloid accumulation. The total surface volume of β-amyloid accumulation in the intervention group was significantly lower than that of the control group (intervention, 122,180,948 μm^3^ [105,854,660–169,063,081]; control, 167,201,016 μm^3^ [139,367,765–193,535,450]; *p* = 0.043). There were no significant differences in the morphological parameters, such as ellipticity and sphericity. Our LSFM study demonstrated notable reductions in β-amyloid, as evidenced by a decrease in total surface volume, in 52-week-old transgenic mice after a 5-week structured swimming program, supporting the notion that even in advanced AD stages, leisure-time voluntary swimming serves as an efficacious intervention for augmenting resistance to pathology.

## 1. Introduction

Recent challenges in pharmacological trials using monoclonal antibodies targeting Alzheimer’s disease (AD) pathology have redirected the focus toward preventing AD through lifestyle interventions. The concept of “resistance” in this context refers to individuals exhibiting lower-than-expected levels of pathology, despite risk factors or genetic predispositions [1]. Among the various modifiable lifestyle interventions identified in the 2020 dementia report by the Lancet Commission, physical activity is particularly crucial, potentially reducing the overall prevalence of dementia by 2%, with outcomes varying based on the type, frequency, intensity, and consistency of the exercise regimen [2].

Numerous observational studies involving nondemented elderly individuals have reported promising associations between leisure-time exercise and a reduced AD risk. In the Honolulu-Asia Aging Study for retired Japanese American men, those who walked less than 0.25 miles per day had a 1.8-fold increased risk of developing dementia compared with those who walked more than 2 miles per day [3]. However, evidence from randomized controlled trials (RCTs) remains insufficient, as no significant differences in cognitive outcomes were found between the exposure and control groups, with beneficial effects observed only in subgroup analyses of individuals over 80 years of age and those with low baseline physical activity levels [4]. Additionally, there is currently insufficient evidence to establish specific clinical guidelines regarding the optimal timing, type, intensity, and duration of exercise throughout the lifespan for effective AD prevention [5]. In previous observational human studies, information about participants’ physical activity history was collected retrospectively through interviews with the participants or their close relatives, a method that limits the accurate application of exercise interventions as intended by the researchers, thereby underscoring the need to study the impact of physical exercise on AD prevention in an experimental animal model, where manipulation and quantification of exercise interventions are relatively easier.

Among various types of exercise, swimming offers advantages in terms of human translatability and is more voluntary and physiological compared to other exercises used in previous studies, such as forced swimming or treadmill running with electric shocks, which are not considered to have true neuroprotective benefits. Additionally, swimming allows for easy quantification by adjusting the duration, interval, frequency, and consistency of the exercise regimen. In our study, we used light-sheet fluorescence microscopy (LSFM) combined with a hydrophilic tissue-clearing technique to clarify the role of voluntary swimming in ameliorating amyloid pathology in a transgenic AD mouse model.

## 2. Results

### 2.1. Effect of the Exercise Intervention on Weight

**Figure 1:**
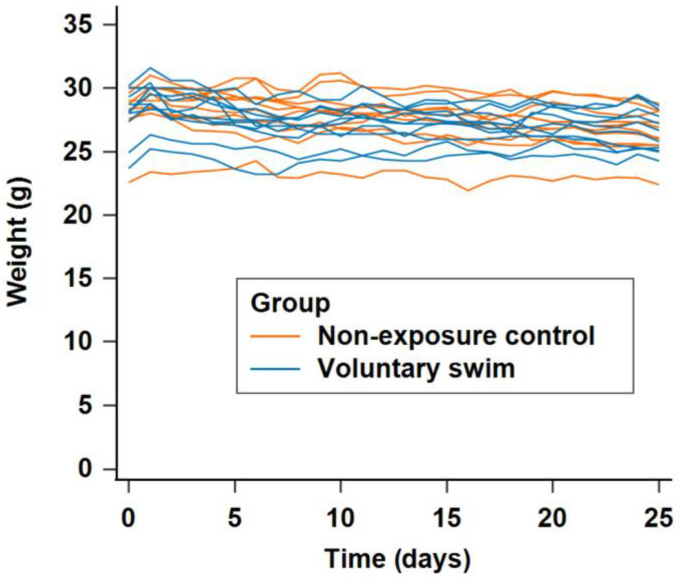
Comparison of the longitudinal trend of daily weight measurements over 5 weeks between mice in the voluntary swim group and the nonexposure control group.

Figure 1 shows the longitudinal trend of daily weight measurements over 5 weeks for the mice in the voluntary swim group and the nonexposure control group. When the difference in weight between the final and baseline measurements was compared, the voluntary swim group presented slightly less weight loss than did the nonexposure control group. However, this difference was not statistically significant (difference between final and baseline weight: voluntary swim group: −0.650 g, nonexposure control group: −0.833 g, *p* = 0.729).

### 2.2. Effect of Voluntary Swimming on Total β-Amyloid Load and 3D Morphological Features in Brain Macro-LSFM Imaging

**Figure 2:**
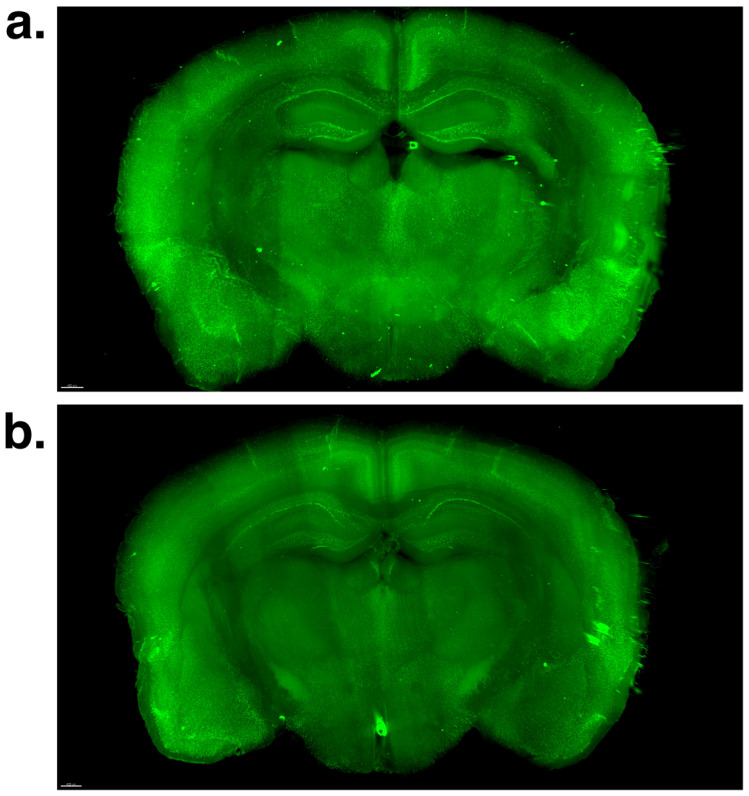
Representative light-sheet illuminating microscope image with hydrophilic tissue clearing and volume immunostaining using thioflavin S (488 nm, green channel) for β-amyloid in the brain. (**a**) the nonexposure control group. (**b**) the voluntary swim group.

Using an advanced light-sheet illuminating fluorescence microscope (LSFM) and hydrophilic tissue-clearing chemical techniques, we conducted a comprehensive investigation into the three-dimensional spatial distribution of β-amyloid accumulation, labeled with thioflavin S (488 nm, green channel), in the brains of the voluntary swim group and the nonexposure control group. In both groups, β-amyloid accumulation was predominantly observed across the cortical regions—including the retrosplenial, posterior parietal association, primary somatosensory, auditory, temporal association, piriform, and amygdalar areas—as well as in the hippocampus (CA1, CA2, CA3, and dentate gyrus), thalamus, and hypothalamus, with particularly severe deposition in the retrosplenial cortex, hippocampus, primary somatosensory cortex, auditory cortex, temporal association cortex, amygdalar area, and piriform area. A visual comparison between the voluntary swim group and the nonexposure control group revealed that the mice subjected to 7 weeks of voluntary swim training presented significantly lower β-amyloid accumulation than the control mice did (Figure 2 and Videos S1 and S2). The light-sheet fluorescence microscopy image of a 51-week-old female C57BL/6 mouse brain visually demonstrates that amyloid is nearly undetectable in this sample, rendering quantification unmeasurable (Supplemental Figure S1).

**Figure 3:**
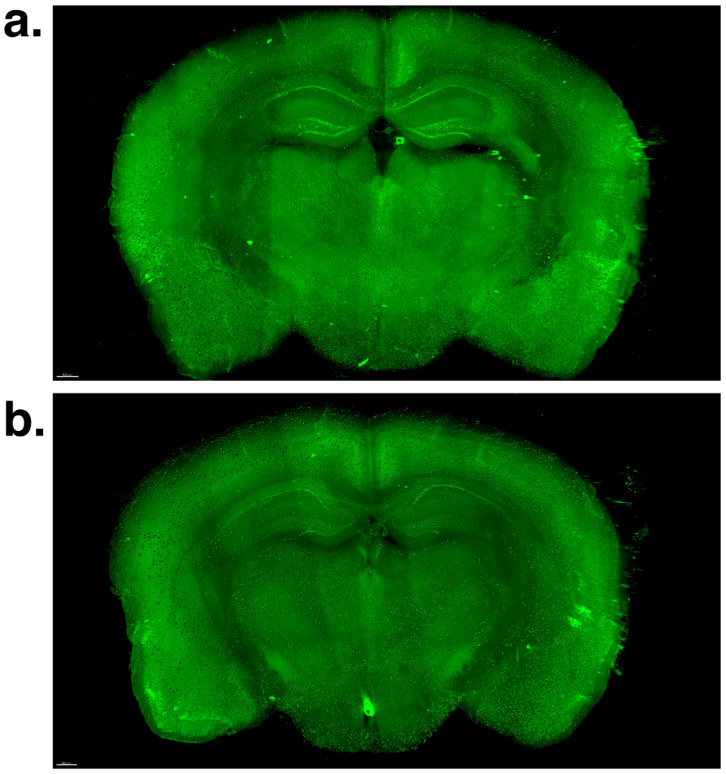
Imaris-based volume rendering surface model for β-amyloid in the brain. (**a**) the nonexposure control group. (**b**) the voluntary swim group.

For our quantitative analysis via Imaris software (Version 7.2.3, Bitplane AG, Zurich, Switzerland), we generated 3D volumetric surface models (Figure 3) to measure various parameters of brain β-amyloid plaques, including the individual plaque size (individual plaque area and volume), variability in individual plaque size (standard deviation of individual plaque volume), total plaque load (total plaque number, surface area, and volume), and morphological shape parameters (individual plaque ellipticity (both oblate and prolate) and sphericity).

**Figure 4:**
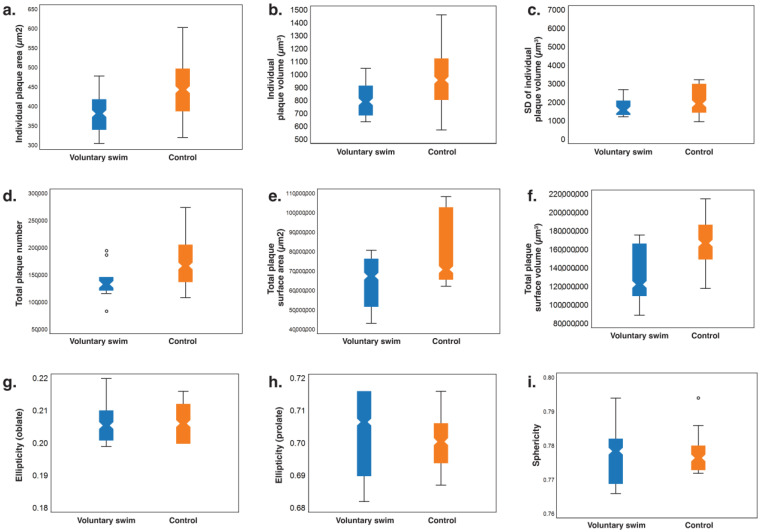
Comparison of the total β-amyloid load and 3D morphological features in the brain Macro-LSFM images between the voluntary swim group (*n* = 10) and the nonexposure control group (*n* = 10). (**a**) individual plaque area (μm^2^). (**b**) individual plaque area volume (μm^3^). (**c**) standard deviation (SD) of the individual plaque volume (μm^3^). (**d**) total plaque number. (**e**) total plaque surface area (μm^2^). (**f**) total plaque surface volume (μm^3^). (**g**) ellipticity (oblate). (**h**) ellipticity (prolate). (**i**) sphericity.

The average total surface volume of β-amyloid accumulation in the brains of the voluntary swim group was significantly lower than that of the nonexposure control group (voluntary swim group, 122,180,948 μm^3^ [105,854,660–169,063,081]; nonexposure control, 167,201,016 μm^3^ [139,367,765–193,535,450]; *p* = 0.043) (Table 1 and Figure 4). Although the total plaque number was slightly lower in the voluntary swim group than in the nonexposure control group, the difference was statistically borderline (voluntary swim group, 133,087 [119,177–167,494]; nonexposure control, 166,724 [135,650–235,889]; *p* = 0.052) (Table 1 and Figure 4). There were no statistically significant differences between the groups in terms of the total surface area (voluntary swim group, 167,459,040 μm^2^ [48,641,450–77,294,670]; nonexposure control, 70,987,024 μm^2^ [65,786,571–102,832,336]; *p* = 0.13), individual plaque area (voluntary swim group, 382.40 μm^2^ [337.47–430.76]; nonexposure control, 443.59 μm^2^ [381.32–506.52]; *p* = 0.063), or individual plaque volume (voluntary swim group, 789.34 μm^3^ [677.58–964.93]; nonexposure control, 961.21 μm^3^ [766.81–1178.85]; *p* = 0.1213). The variability in the individual plaque size, measured by the standard deviation of the individual plaque volume, did not differ significantly between the groups (voluntary swim group, 1608.693 μm^3^ [1340.4418–2406.660]; nonexposure control, 1940.021 μm^3^ [1417.318–3022.223]; *p* = 0.315). Additionally, there were no statistically significant differences in the morphological shape parameters of individual amyloid particles, such as ellipticity (oblate: voluntary swim group, 0.206 [0.201–0.212]; nonexposure control, 0.206 [0.200–0.213]; *p* = 0.939), ellipticity (prolate: voluntary swim group, 0.707 [0.686–0.716]; nonexposure control, 0.701 [0.692–0.711]; *p* = 0.675), or sphericity (voluntary swim group, 0.779 [0.769–0.783]; nonexposure control, 0.777 [0.773–0.783]; *p* = 0.791).

## 3. Discussion

Compared with nonexposure controls, light-sheet fluorescence microscopy (LSFM) imaging combined with hydrophilic tissue clearing techniques revealed that the voluntary swim exercise group exhibited reduced amyloid accumulation, measured as the total amyloid surface volume. Our findings support the notion that even in the advanced stages of Alzheimer’s disease, leisure-time voluntary swimming serves as an effective intervention for mitigating amyloid pathology by increasing resistance to the disease.

Physical exercise is well-established to reduce β-amyloid plaques in the brain through a variety of biomolecular mechanisms, as demonstrated in multiple studies [6,7,8]. For instance, aerobic exercise increases nitric oxide (NO) bioavailability, which supports the glymphatic clearance of amyloid-β and hyperphosphorylated tau aggregates, thereby preventing astrocyte activation and the release of proinflammatory cytokines that damage blood vessels in aged mice [6]. In addition, exercise enhances glymphatic influx in awake, active young mice, facilitating the clearance of pathological aggregates [7]. Exercise also improves cerebral blood flow, reduces cerebral amyloid angiopathy, and enhances endothelial function [9]. Furthermore, aerobic exercise induces the expression of biomolecules such as vascular endothelial growth factor (VEGF), brain-derived neurotrophic factor (BDNF), and insulin-like growth factor 1 (IGF-1) in endothelial cells, which mediate cognitive protection. BDNF promotes neuronal proliferation, IGF-1 regulates neural and angiogenic processes, and VEGF supports the survival and growth of blood vessels [10]. These findings highlight the multifaceted mechanisms by which exercise exerts its neuroprotective effects.

Voluntary exercise has been shown to be more beneficial than forced exercise for preventing dementia and maintaining physiological stability during exercise sessions. In a previous comparison study involving 8-week regimens of forced and voluntary wheel running exercises in 34 female Long-Evans rats, both groups covered the same total distance; however, the voluntary group exhibited higher speeds and shorter exercise durations than did the forced exercise group [11]. When the survival of bromodeoxyuridine (BrdU)-labeled hippocampal progenitor cells in the dentate gyrus was assessed, both types of exercise increased the number of surviving cells, with the forced exercise group showing significantly more surviving cells than the voluntary exercise group [11]. However, the forced exercise group also exhibited increased anxiety-like behavior and emotional defecation in the open-field test, suggesting that forced exercise may acutely elevate corticosterone levels more than voluntary wheel running does [11,12]. Voluntary and forced exercise differentially affect monoamine neurotransmitters [11], hippocampal BDNF, and synapsin-1 expression [12], as well as cognition and behavior [13,14]. Previous studies have shown that mice subjected to forced swim apparatus interventions exhibit low brain BDNF levels and increased blood lactate levels due to apnea and the stress from the possibility of drowning [15]. Therefore, we selected voluntary swimming in a wavy water tank as our intervention, as this protocol maintains physiological stability during exercise sessions and does not pose a significant threat to the animals.

Our analysis first demonstrated that 52-week-old transgenic AD mice subjected to a 5-week regimen of voluntary swimming for up to 30 min daily in 15 cm deep, wavy water exhibited reduced β-amyloid accumulation, as measured by total amyloid surface volume in the whole brain, compared with nonexposed controls. The present study not only quantifies the total amyloid load in the brain but also provides detailed measurements of individual plaque areas and volumes, variability in individual plaque volume, and morphological features such as ellipticity and sphericity, which were challenging to assess with earlier techniques. Traditional experimental techniques used in previous research involve pathology spot counting in thin two-dimensional (2D) sections of localized subregions, which limits the assessment of the three-dimensional (3D) spatial distribution of amyloid-beta (Aβ) particles within whole, intact brain structures. In contrast, Macro-LSFM, combined with tissue-clearing technologies, offers an expansive field of view without compromising spatial resolution, enabling imaging of entire brain structures while preserving their 3D integrity and providing comprehensive insights into the 3D spatial distribution of complex neuropathological networks within the fully transparent brain [16,17,18,19]. In addition to the significant reduction in total amyloid surface volume, the swim exercise regimen slightly reduced the total plaque number, total surface area, and area or volume of individual plaques. The reduction in total plaque number was more pronounced than the reduction in the area or volume of individual plaques, although these differences were not statistically significant because of the small sample size. Interestingly, the voluntary swimming exercise regimen did not influence the morphological features of each individual amyloid particle, such as their ellipticity and sphericity.

Various factors that could influence our results include the age and sex of the animals; the environmental conditions under which the swimming took place, such as the water depth, temperature, and wave intensity; the duration of daily swimming; the intervals between swimming and rest periods; and the overall duration of the swimming regimen. In our study, we adapted a swimming protocol from previous investigations involving voluntary swimming programs [6,20]. In a study with 11–12-week-old type 2 diabetic rats, a 4-week voluntary swimming regimen of up to 60 min per day was found to reduce depression-like behavior by mitigating inflammation [6]. Given the differences in physical conditions, such as weight and basal metabolic rates, and neurobiological systems between AD transgenic mice and nondemented rats, we modified the swimming protocol from previous studies by reducing the daily swimming duration to 30 min and extending the total period to 5 weeks. Considering the heightened sensitivity of AD transgenic mice to new stimuli, this modification aimed to balance the prevention of stress and health issues in mice with the efficacy of exercise on AD pathology.

Given previous evidence on the moderating effects of aging on the cognitive response to exercise in older populations [21], the advanced age of the 52-week-old transgenic AD mice may have contributed to the effectiveness of the voluntary swimming exercise intervention in reducing the total amyloid surface volume in the brain. According to the 2018 Health and Human Services Physical Activity Guidelines for Americans Advisory Committee, there is moderate-to-strong evidence that both acute and long-term moderate- to vigorous-intensity physical activity interventions benefit brain structure and function, as well as cognitive functioning, during late periods of life, i.e., over 50 years of age [5]. In a previous study involving 7-month-old 5xFAD mice, long-term voluntary physical exercise increased the number of glial fibrillary acidic protein (GFAP)-positive astrocytes and modulated the reactive astrocyte state, potentially linking astrocytic BDNF and PSD-95 to improved cognition in the hippocampus of 5xFAD mice [22]. Various circulating biomolecules, such as brain-derived neurotrophic factor (BDNF), vascular endothelial growth factor (VEGF), and insulin-like growth factor (IGF)-1, may play different roles in childhood versus older adulthood, potentially influencing sensitivity to exercise [21]. These biomolecules, which mediate exercise-induced cognitive improvement, become dysregulated with aging [21,23]. In a randomized, community-based clinical trial involving 132 cognitively normal individuals aged 20–67 years, executive function improved significantly in the aerobic exercise group, with the effect moderated by age (β = 0.018 SD/year; *p* = 0.028), with increases of 0.228 SD at age 40 years (95% CI, 0.007–0.448) and 0.596 SD at age 60 years (95% CI, 0.219–0.973) [23]. The effect of aerobic exercise on executive function was more pronounced with increasing age, suggesting that it may mitigate age-related cognitive decline [23]. Consequently, older individuals who are deficient in these biomolecules may experience greater cognitive benefits from exercise than those with normal levels [21].

A limitation of our study is that our outcomes focused solely on resistance to pathology rather than on neurodegenerative outcomes, such as cortical thickness or hippocampal volume, or cognitive function outcomes. Furthermore, thioflavin-S staining, a widely used method for detecting Aβ plaque-like features, is limited by its non-specific binding to other β-sheet–rich structures, such as *p*-tau aggregates. This necessitates caution when using Thioflavin-S as a probe for detecting Aβ in transgenic mouse models of Alzheimer’s disease. Despite these limitations, Thioflavin-S was selected over anti-Aβ antibodies in this study due to the inherent challenges of antibody penetration in thick, whole-organ samples. The small-molecule properties of Thioflavin-S facilitate consistent and uniform staining in such preparations. However, prior studies have shown that Thioflavin-S-labeled protein aggregates in the cortical and hippocampal regions of 5xFAD mice do not always correspond to anti-Aβ antibody staining [24]. Furthermore, higher concentrations of the dye have been associated with non-specific binding and red-shifted fluorescence, highlighting the need for careful optimization of staining protocols [25]. These findings underscore the importance of using complementary approaches, such as co-labeling with Aβ-specific antibodies, to ensure a more accurate and comprehensive assessment of amyloid pathology [25]. Additionally, our study does not investigate the molecular mechanisms by which exercise may influence β-amyloid plaque deposition, nor does it evaluate its effects on other key pathologies, such as tau and neuroinflammation. Future research should comprehensively assess the impact of voluntary swimming exercise on multiple aspects of Alzheimer’s disease pathology, including amyloid, tau, neuroinflammation, brain structure, and cognition. We also quantified the amyloid pathology burden across the whole brain but did not use automated mapping techniques for subregional quantification within specific anatomical subregions. Furthermore, our study evaluated the impact of a 5-week swimming exercise regimen on amyloid pathology in 52-week-old transgenic AD mice. Future studies should compare the effects of initiating exercise at earlier stages and maintaining it for longer durations to identify the optimal timeframes for maximizing protective effects on pathology resistance, and these comparisons should be verified in well-designed RCTs using a life-course approach.

## 4. Materials and Methods

### 4.1. Animal Model

For the current investigation, we assembled an experimental cohort of twenty 52-week-old female C57BL/6-Tg (NSE-hAPPsw) Korl mice, commonly referred to as the APPsw model, in which the onset of dementia is accelerated by the overexpression of the Swedish amyloid precursor protein (APPsw; Lys670→Asn, Met671→Leu) within brain tissue. Six 51-week-old female C57BL/6 mice were used as non-AD negative controls. All mice were housed in a temperature-controlled environment (22 ± 2 °C, 55 ± 10% relative humidity) with a 12-h light/dark cycle, provided ad libitum access to standard rodent feed and water, and accommodated in standard polycarbonate cages with autoclaved wood shavings and enrichment materials. All animal experiments were approved by the Dankook Institutional Animal Care and Use Committee and conducted in accordance with their guidelines and regulations (DKU-23-041).

### 4.2. Voluntary Swimming Exercise Regimen

The swimming exercise, on the basis of previous research [6], was conducted using a stainless-steel water tank (Φ900 × H450 mm/550 mm) filled with fresh warm water at a controlled temperature (32 ± 1 °C) and equipped with three identical wave generators (Jebao, Zhongshan, China) arranged in an equilateral triangle to ensure that the mice were compelled to swim and unable to merely float. Twenty transgenic AD mice were randomly divided into two subgroups: voluntary swim (*n* = 10) and nonexposure control (*n* = 10). The exercise regimen involved swimming 5 days per week for 5 weeks, with each session consisting of a 10-min swim followed by a 10-min rest. There were two phases (adaptation and training) in the swimming exercise program [20,26,27]. During the first week of the adaptation phase, the mice were gradually acclimated to a shallow water depth (5 cm) for 10-min sessions daily. This gradual acclimation process allowed the mice to adjust to the aquatic environment, minimizing stress-related physiological and behavioral responses that could otherwise confound the effects of the exercise regimen. Starting in the second week of the training phase, the water depth was increased to 15 cm, and the swimming time was extended to 20 min (two 10-min sessions). In the third to fifth weeks, the water depth remained at 15 cm, and the swimming time was increased to 30 min (three 10-min sessions), with 10-min rest intervals between swims. After each swim session, the mice in the voluntary swim group were carefully removed from the tank, dried with paper towels, and kept warm with an infrared lamp during the 10-min rest intervals. The nonexposure control group was placed in an identical empty tank and allowed free movement for the same duration [20,26,27]. By utilizing identical tank setups without active wave generators, we allowed the mice to engage in their natural movements and exploratory behaviors in non-stimulated environments, thereby ensuring that both groups experienced comparable levels of environmental stimulation except for the enforced swimming activity. This design enabled any observed differences between the groups to be more confidently attributed to the voluntary swimming exercise, thereby minimizing potential confounding factors related to baseline activity levels.

### 4.3. Hydrophilic Clearing Process and Volume Immunostaining for Amyloid-Beta (Aβ) in Brain Samples

According to the protocols established by the Institutional Animal Care and Use Committee (IACUC), mice were euthanized with isoflurane. Subsequently, transcranial perfusion with 1X phosphate-buffered saline (PBS) (Gibco, Thermo Fisher Scientific, Waltham, MA, USA) was performed to effectively remove blood from the brain vasculature. The brain tissues were then fixed with a 4% paraformaldehyde (PFA) solution (Sigma-Aldrich, St. Louis, MO, USA) in PBS. Following fixation, the brains were carefully dissected and subjected to overnight postfixation at 4 °C in 4% PFA. A 1 mm sagittal section was then dissected, specifically targeting the CA1 hippocampal region, with coordinates relative to the bregma: −2 mm anterior/posterior, ±1.8 mm lateral/medial, and −1.5 mm dorsal/ventral. Tissue clearing was achieved using a hydrophilic Binaree Tissue Clearing Solution Kit (HRTI-001, Binaree, Daegu, Korea). PFA was removed by three washes with 1X PBS at 4 °C, each lasting 20 min. The samples were then submerged in 10 mL of starting solution and incubated at 37 °C for 48 h while shaking at 50 rpm. The solution in each tube was then replaced with 10 mL of Tissue Clearing Solution A, and the incubation was repeated. Each tube was rinsed four times with distilled water at 4 °C, with each wash lasting 30 min. The solution was then substituted with 3 mL of Tissue Clearing Solution B, and the samples were incubated again at 37 °C for 12 h while shaking at 50 rpm. To visualize amyloid-beta (Aβ) plaques, the samples were treated with thioflavin S, which emits at 488 nm. The plaques were stained with a 1% thioflavin S solution in 50% ethanol, incubated at 25 °C, shaken at 50 rpm for 15 min, and then washed three times with 1X PBS at 4 °C, each wash lasting 20 min. Finally, the samples were incubated in 6 mL of mounting and storage solution (SHMS-060, Binaree, Daegu, Korea) at 37 °C for 48 h while shaking at 50 rpm to match the high refractive index of the tissue and render the tissues transparent. Mounted samples were stored in the dark at room temperature until light-sheet fluorescence microscopy (LSFM) imaging.

### 4.4. Light-Sheet Illuminating Fluorescence Microscope (LSFM) Imaging Acquisition and Imaris Software-Based Automated Quantitative Analysis of 3D Volumetric Surface Model Generation

**Figure 5:**
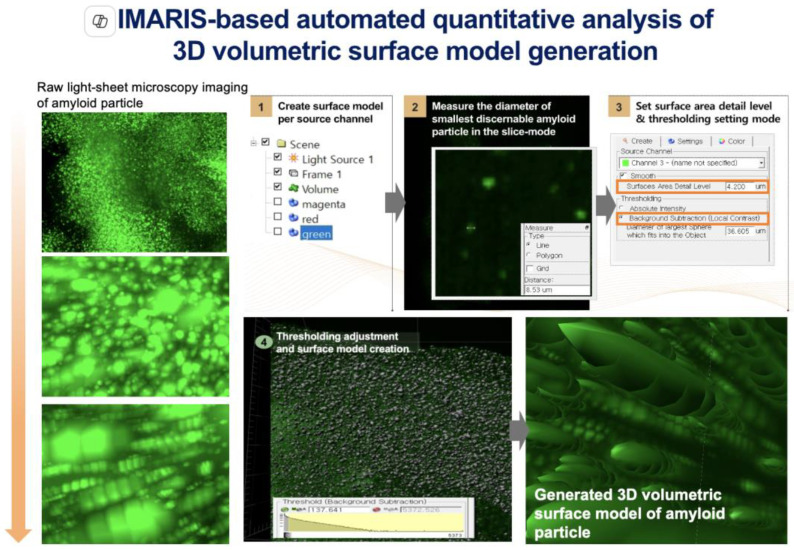
Workflow of the analytical pipeline for generating the Imaris-based automated 3D volumetric surface model of β-amyloid plaques.

High-resolution Macro-LSFM imaging of cleared brain samples was performed using a Zeiss Lightsheet Z.1 microscope system (Carl Zeiss Meditec, Inc., Oberkochen, Germany) (Figure 5). A quintuple magnification objective lens (NA = 0.16) with a 0.75× zoom factor was used for optical image acquisition. The LSFM apparatus was calibrated with an excitation laser wavelength of 488 nm for the detection of amyloid-beta (Aβ) deposition in the brain. Single-illumination mode was employed to increase the signal-to-noise ratio and overall image clarity. Following acquisition, the raw data were transcribed into CZI (Carl Zeiss Image) files for analytical processing. Quantitative image evaluation was performed using Imaris software (Version 7.2.3, Bitplane AG, Zurich, Switzerland). The dimensions of the three-dimensional volumetric data (length, width, and height) were each downscaled to 25% of their original size to optimize computational efficiency during image analysis. For the 488 nm laser wavelength used for amyloid-beta (Aβ) detection, a computational surface model was generated using the Surface Creation Wizard embedded within the Imaris software environment. The minimum discernible feature size was set at 4.0 μm, corresponding to the smallest detected diameter of the targeted Aβ markers within the slice-mode observations. Local contrast enhancement was applied for background subtraction to segregate the true signal from extraneous noise. An automated thresholding approach defined the largest permissible feature size in the extraneous background, which was then manually refined to ensure that the constructed surfaces accurately replicated the morphological attributes of the cellular markers while minimizing aberrant noise. Statistical validation was conducted by applying a 95% confidence interval to the voxel dimensions, accurately determining the volumetric characteristics of the surface models. Volumetric data extraction was facilitated through the “Statistics” functionality within the “Surpass” tab of the Imaris interface, with the “Volume” metric specifically selected from the available measurement parameters.

### 4.5. Statistical Analysis

Data are presented as the medians [IQRs] unless specified otherwise. A Mann–Whitney test was used to determine differences in body weight between final and baseline measurements, as well as in quantitative parameters representing total brain β-amyloid load and 3D morphological features of β-amyloid between the AD and control mouse groups. A two-sided *p* value <0.05 was considered statistically significant. All the statistical analyses were performed using SAS version 9.4 (SAS Institute; Cary, NC) and R 4.2.2 (R Foundation for Statistical Computing, Vienna, Austria) (https://cran.r-project.org/, accessed on 30 January 2025).

## 5. Conclusions

In conclusion, by utilizing advanced Macro-LSFM imaging with hydrophilic tissue-clearing techniques, this study comprehensively investigated the three-dimensional (3D) spatial distribution of amyloid-beta (Aβ) pathology in the chemically cleared brains of 52-week-old transgenic AD mice, quantifying the total amyloid load in the brain and providing detailed measurements of the morphological features of individual amyloid particles, which were challenging to assess with earlier techniques. Our findings lend credence to the notion that even in advanced AD stages, leisure-time voluntary swimming physical activity serves as an efficacious modifiable lifestyle intervention for augmenting resistance to AD pathology.

## Figures and Tables

**Table 1:** Comparison between the voluntary swim group and nonexposure control mouse groups.

Parameters	Voluntary Swim (*n* = 10) Median [IQR]	Nonexposure Control (*n* = 10) Median [IQR]	*p*
Individual plaque area, µm^2^	382.40 [337.47–430.76]	443.59 [381.32–506.52]	0.063
Individual plaque volume, µm^3^	789.34 [677.58–964.93]	961.21 [766.81–1178.85]	0.123
SD of individual plaque volume, µm^3^	1608.693 [1340.4418–2406.660]	1940.021 [1417.318–3022.223]	0.315
Total plaque number	133,087 [119,177–167,494]	166,724 [135,650–235,889]	0.052 *
Total plaque surface area µm^2^	67,459,040 [48,641,450–77,294,670]	70,987,024 [65,786,571–102,832,336]	0.130
Total plaque surface volume, µm^3^	122,180,948 [105,854,660–169,063,081]	167,201,016 [139,367,765–193,535,450]	0.043 *
Ellipticity (oblate)	0.206 [0.201–0.212]	0.206 [0.200–0.213]	0.939
Ellipticity (prolate)	0.707 [0.686–0.716]	0.701 [0.692–0.711]	0.675
Number of vertices	23.487 [21.811–29.675]	25.702 [22.356–29.021]	0.578
Sphericity	0.779 [0.769–0.783]	0.777 [0.773–0.783]	0.791

Abbreviations: IQR: interquartile range. Notes: * *p* < 0.05 was considered statistically significant.

## Data Availability

All research data and computer codes are available from the corresponding author upon request.

## Supplementary Materials

The following supporting information can be downloaded at: https://www.mdpi.com/article/10.3390/ijms26031249/s1.
