# Supplementary material for: Light-Sheet Fluorescence Imaging Reveals Three-Dimensional Amyloid Burden Reduction Following Five Weeks of Swimming Exercise in Alzheimer’s Mouse
Source: Int J Mol Sci. 2025 Jan 31;26(3):1249. doi: 10.3390/ijms26031249 (PMC11818873; doi:10.3390/ijms26031249)
Supplement: Supplementary file 1 [file ijms-26-01249-s001.zip › ijms-3439949-supplementary/ijms-3439949-supplementary.pdf]

Supplemental information

Light-sheet fluorescence imaging reveals three-dimensional amyloid burden reduction following five weeks of swimming exercise in Alzheimer's mouse brain

Table of contents

Supplemental method: Light-sheet fluorescence microscope (LSFM) imaging acquisition and Imaris software-based automated quantitative analysis of 3D volumetric surface model generation 3

Supplemental figure 1 10

eReferences 13

Supplemental methods: Light-sheet fluorescence microscope (LSFM) imaging acquisition and Imaris software-based automated quantitative analysis of 3D volumetric surface model generation

High-resolution LSFMimaging of ex vivo brain and eyeball samples was conducted using a Zeiss Lightsheet Z.1 microscope system (Carl Zeiss Meditec, Inc., Oberkochen, Germany). A quintuple magnification objective lens (NA = 0.16) with a 0.75x zoom factor was employed for optical image acquisition. The LSFM apparatus was calibrated with three excitation laser wavelengths specifically selected for the targeted cellular markers: 488 nm for the detection of Aβ aggregates. Single-illumination mode was used to increase the signal-to-noise ratio and overall image clarity. Following acquisition, the raw data were transcribed into CZI files for analytical processing. The quantitative image evaluation was executed using Imaris software (Version 7.2.3, Bitplane AG, Zurich, Switzerland). Native CZI files were converted into an Imaris-compatible data schema for further analysis. The dimensions of the three-dimensional volumetric data (length, width, and height) were each downscaled to 25% of their original extents, optimizing computational efficiency during image analysis. Z-stacks were opened in Imaris are automatically reconstructed into a multi-channel 3D model during input into Imaris. To designate a computational surface model of individual amyloid particle of interest, the Surface creation tool was used to generate a ROI. In the Surface creation wizard within the Imaris software environment, the source channel was set to green channel (488 nm) for amyloid-beta (Aβ).

To ensure reproducibility and minimize user bias, we employed the automated thresholding functionalities integrated into the Imaris software, which algorithmically determined thresholds using standardized parameters without manual adjustments. The initial threshold value was automatically calculated using the algorithm proposed by Ridler et al. (1978).[1] These functionalities, including Gaussian filtering, background subtraction, and auto-local thresholding, were designed to standardize the segmentation process and ensure an objective, reproducible workflow.[2] Gaussian filtering was applied voxel by voxel during preprocessing to enhance the signal-to-noise ratio, reduce noise, and smooth local intensity variations while preserving meaningful structural features. [3-4] Using a convolution operation, each voxel's intensity was replaced by a weighted average of its own and

neighboring values, governed by a Gaussian function.[3-4] To generate surface borders around individual amyloid particles, surface smoothing was performed using a grain size of 4 μm, corresponding to the smallest discernible diameter of targeted cellular markers in slice-mode observations. Background subtraction (local contrast) in Imaris isolated foreground particles from the background by calculating a variable baseline intensity for each voxel and subtracting it from the original intensity.[2] Positive values were retained, while negative values were set to zero, effectively isolating the signal of interest.[2] By reducing background artifacts, this process enhanced the signal-to-noise ratio and ensured that only meaningful structures were retained for analysis.[2] The auto-local thresholding feature significantly improved segmentation accuracy by dynamically adjusting intensity thresholds to reflect the local characteristics of the image.[5] The Niblack method, which calculates thresholds based on the local mean and standard deviation, was particularly effective for images with uneven illumination or noisy backgrounds.[5] Unlike global thresholding, which applies a uniform intensity cutoff across the entire image, auto-local thresholding minimized within-group variance among pixel clusters to determine optimal thresholds.[6-7] This method reduced the misclassification of background pixels with intermediate gray-scale values as foreground, preserving the original morphology of target clusters and yielding well-defined, accurate representations.[8] Through these processes, the size and shape of the generated surfaces directly mapped the intensity distribution of thioflavin-specific Aβ labeling within the eyeball and brain, as detected by Imaris. While automated thresholding was the primary method, a subsequent manual correction step addressed errors such as misidentifying noncellular particles (e.g., fibrous dust), creating a refined semi-automated process that balanced accuracy and consistency. Furthermore, to minimize bias, all data were anonymized, and analysts performed thresholding and manual corrections while blinded to the animals' genotypes, PET results, and experimental conditions. Finally, volumetric data extraction was facilitated through the "Statistics" functionality within the "Surpass" tab of the Imaris interface, with the "Volume" metric specifically selected from the available measurement parameters. Statistical validation was performed by applying a 95% confidence interval to the voxel dimensions, accurately determining the volumetric characteristics of the surface models.

Supplemental figure

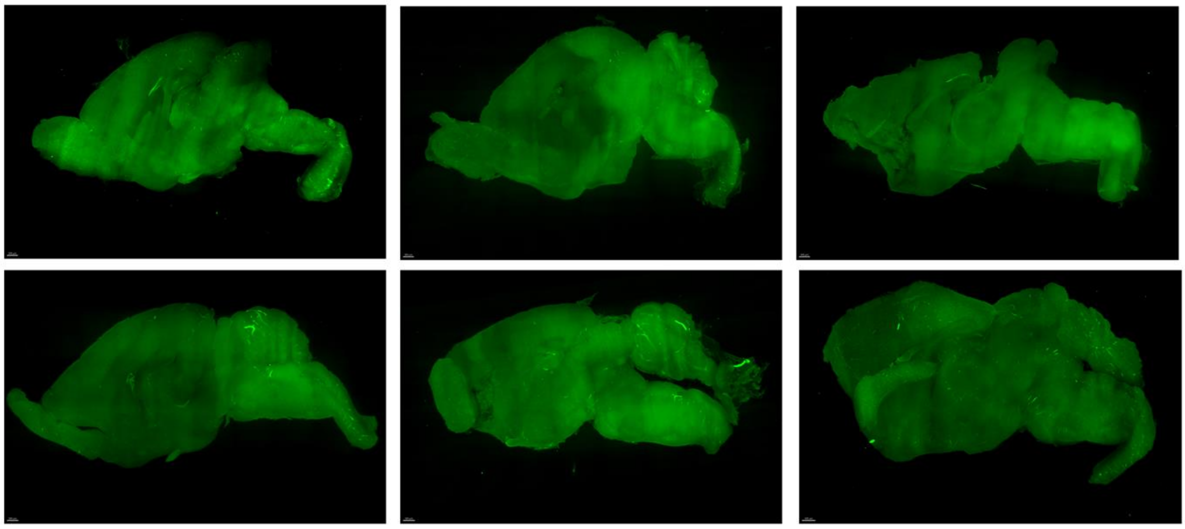

Supplemental Fig. 1 Light-sheet fluorescence microscopy image of a 51-week-old female C57BL/6 mouse brain, prepared using hydrophilic tissue clearing and volume immunostaining with thioflavin S (488 nm, green channel)

References

1. Ridler TW, Calvard S. Picture thresholding using an iterative selection method. IEEE Trans Syst Man Cybern 1978;8:630-632.
2. Bitplane. Imaris Reference Manual. In: Bitplane (ed). Concord, MA; 2017.
3. Oberholzer M, Ostreicher M, Christen H, Brühlmann M. Methods in quantitative image analysis. Histochem Cell Biol 1996;105:333-355.
4. Pham DL, Xu C, Prince JL. Current methods in medical image segmentation. Annu Rev Biomed Eng 2000;2:315-337.
5. Otsu N. A threshold selection method from gray-level histograms. Automatica 1975;11:23-27.
6. Niblack W. An introduction to digital image processing: Strandberg Publishing Company; 1985.
7. Stockman G, Shapiro LG. Computer vision: Prentice Hall PTR; 2001.
8. Chaubey AK. Comparison of the local and global thresholding methods in image segmentation. World Journal of Research and Review 2016;2:1-4.
